# Updates on Selective Brain Hypothermia: Studies From Bench Work to Clinical Trials

**DOI:** 10.3389/fneur.2022.899547

**Published:** 2022-05-06

**Authors:** Xiaoyu Wang, Alexandra Wehbe, Shawn Kaura, Naveed Chaudhry, Xiaokun Geng, Yuchuan Ding

**Affiliations:** ^1^Department of Luhe Institute of Neuroscience, Capital Medical University, Beijing, China; ^2^Department of Neurology, Beijing Luhe Hospital, Capital Medical University, Beijing, China; ^3^Department of Neurosurgery, Wayne State University School of Medicine, Detroit, MI, United States; ^4^Lake Erie College of Osteopathic Medicine at Seton Hill, Greensburg, PA, United States

**Keywords:** acute ischemic stroke (AIS), selective hypothermia, rodents, primates, clinical translation

## Abstract

Thrombectomy or thrombolysis are the current standards of care for acute ischemic stroke (AIS), however, due to time constraints regarding operations and a multitude of contraindications, AIS remains one of the leading causes of death and chronic disability worldwide. In recent years, therapeutic hypothermia has been explored as an adjuvant therapy for AIS treatment and has shown potential to improve outcomes in patients with AIS. In particular, selective therapeutic hypothermia has shown to markedly reduce infarct volumes and have neuroprotective effects, while also minimizing many systemic side effects seen with systemic therapeutic hypothermia. Both preclinical and clinical trials have demonstrated that selective therapeutic hypothermia is a safe and feasible therapy for patients who have suffered an AIS. In this review, we summarize the current update on selective hypothermia through major studies that have been conducted in rodents, large animals, and clinical trials, and briefly discuss the prospects of selective hypothermic research. We hope this review helps facilitate the exploration of other possible adjuvant treatment modalities in the neuroprotection of ischemic stroke, whether upon symptom onset or after vascular recanalization.

## Introduction

Fifteen million people suffer a stroke annually, making it the leading cause of acquired disability and second leading cause of death worldwide (1). Acute Ischemic Stroke (AIS) treatment and rehabilitation is an astronomical burden on healthcare systems despite its limited efficacy. AIS has a striking 30-day case fatality rate of 16–23% and leaves up to 50% of survivors chronically disabled (2–4). Vascular recanalization, including thrombolysis and thrombectomy, has been proven to be effective in ideal circumstances. However, because of the narrow therapeutic window for vascular recanalization, AIS morbidity and mortality rates remain high (5). Thus, there is an urgent need to explore alternative and adjuvant treatments (6, 7).

Therapeutic Hypothermia (TH) is a novel treatment for AIS that has been heavily studied and proven to be one of the most effective adjunctive treatments for AIS in Pre-clinical models (8–12). TH intentionally lowers the body temperature to reduce neurologic damage. TH serves as a neuroprotectant by attenuating numerous metabolic and molecular pathways involved in the progression of AIS such as suppressing free radical production, reducing production of inflammatory mediators, modifying ischemia-mediated calcium influx, and reducing blood brain barrier disruption (13). There is growing hope that TH could substantially reduce AIS morbidity and mortality.

TH may be done systemically, by whole body cooling, or selectively, by lowering brain temperature while maintaining core temperature. Systemic TH is done through external surface cooling (e.g., using air blankets, cold saline and alcohol washes, water mattresses, and ice packs), infusions of cooled saline into the veins, or special transvenous endovascular cooling devices. Studies on systemic TH have provided valuable insight on the safety and practicality of this treatment. However, systemic TH poses risks of serious side effects such as hypotension, cardiac arrhythmia, and pneumonia due to its whole-body cooling effects (14–20). Further, previous studies on systemic TH have demonstrated great variability in time-to-target temperature, some up to several hours. Since the neuroprotective window for AIS is usually only 4.5 h, achieving target temperature through systemic methods may not be a practical therapeutic method. However, more recent systemic TH studies showed that, compared with surface cooling techniques that may take over 4 h, intravascular cooling (*via* the inferior vena cava) combined with intravenous tissue plasminogen activator or a drug cocktail only takes about 1 h to reach target temperature (21, 22).

Selective TH can be achieved through external surface cooling of the head and neck intranasally via devices that pump coolant mists or cooled air into the nasal cavity, endovascular cold saline infusion via intraarterial catheters, or more directly into the ischemic region using intracranial catheters (23–25). Selective TH minimizes systemic side effects seen with systemic TH, such as systemic hypothermia and pneumonia, and reaches target temperatures faster than systemic methods using less fluid than required for systemic cooling (24). Selective TH has made major breakthroughs in rodent and large animal models and shows great clinical promise as an adjuvant treatment for AIS.

In recent years, researchers have made great progress in demonstrating the neuroprotective effects of selective TH in patients with AIS. From rodents, primates, to clinical studies, it has been shown that selective TH is a viable adjunctive technique both Pre- and Post-reperfusion. In this review, we introduce the latest progress of selective TH research, laying the foundation for its transition to clinical practice.

## The Concept of Selective Hypothermia and Animal Research

### Rodent Stroke Models

Rodent models were the preferred models for early selective TH studies after AIS. Many studies demonstrated that selective TH significantly reduced the volume of infarction in the experimental group compared to the control group with normal body temperature (Table 1). However, these studies varied in the timing of selective TH induction, as well as varying degrees of hypothermia and ischemic injury.

**Table 1:** Studies on rodents.

**Author**	**Infusate/Volume**	**Infusion rate**	**Infusion time**	**Time to TT**	**Brain temp**	**Core body temp**	**Infarct volume**	**Functional outcome**
Ding et al. (26)	Saline 23 °C (7 ml)	2 ml/min	3–4 min	3–4 min	32–33°C	——	Reduced	Improved
	Saline 37°C (7 ml)	2 ml/min	3–4 min	——	37°C	——	Reduced	Improved
Ding et al. (27)	Saline 37°C (6 ml)	2 ml/min	3 min	——	——	——	——	——
Kurisu et al. (14)	Saline 10°C (4.8–6.2 ml)	0.32–0.41 ml/min	15 min	<5 min	Cortex 34.8°C Striatum 35.4°C	>36°C	Reduced	Improved
Zhao et al. (28)	Saline 20°C (6 ml)	0.6 ml/min	10 min	<10 min	Cortex 32.8–33.2°C Striatum 33.2–33.3°C	>37°C	Reduced	Improved
Ding et al. (29)	Saline 20°C (6 ml)	0.6 ml/min	10 min	<5 min	Cortex 33.4°C Striatum 33.9°C	>36°C	Reduced	Improved
Li et al. (30)	Saline 20°C (6 ml)	0.6 ml/min	10 min	——	——	——	Reduced	Improved
Luan et al. (25)	Saline 20°C (6 ml)	0.6 ml/min	10 min	<5 min	Cortex 33.4°C Striatum 33.9°C	>36°C	——	——
Ji et al. (31)	Saline 10°C (7.5 ml)	0.25 ml/min	Interrupted pattern	6 min	34.6°C	37°C	Reduced	Improved

*TT, Target Temperature*.

**Figure 1:**
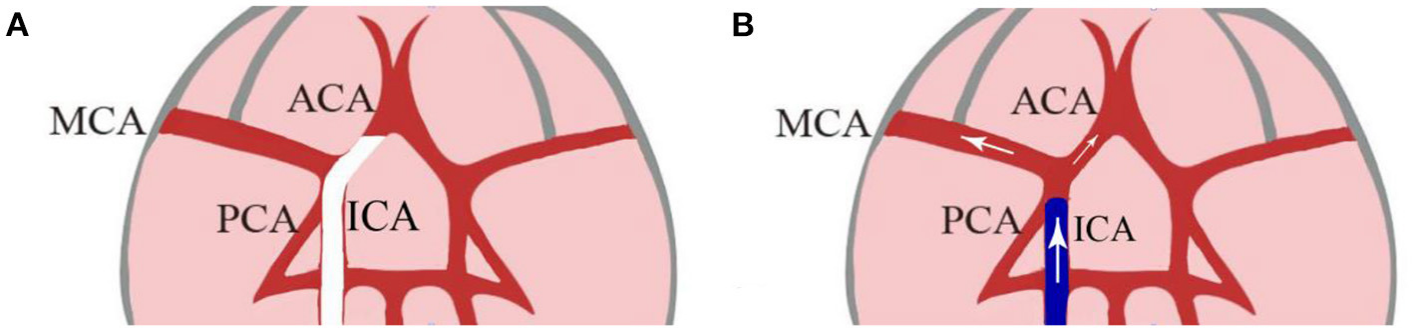
A hollow catheter lodges in the narrow proximal ACA and blocks the MCA at its origin **(A)**. After 2 h of occlusion, saline was flushed into the junction of the MCA and ACA as the hollow filament is withdrawn 1 to 2 mm from the origin of MCA **(B)**. [Modified from Ding et al. (26)]. ACA, anterior cerebral artery; MCA, middle cerebral artery; ECA, external carotid artery; ICA, internal carotid artery.

Intra-arterial cold saline infusion has been the core method used in the study of selective TH for preclinical AIS rodent models. IA-CSI was a novel method of selective TH evaluated by Ding et al. in 2002 in an effort to localize TH to minimize its systemic side effects (26). Ding et al. induced transient middle cerebral artery occlusion (tMCAO) using an intraluminal filament, and subsequently infused 7 ml of 23°C or 37°C isotonic saline into the ischemic region at a rate of 2 ml/min (Figure 1). Both 23°C and 37°C saline infusion were found to significantly reduce infarct volumes and improve functional neurologic preservation 48 h Post- reperfusion. A follow-up study conducted by Ding et al. in 2003 (27) demonstrated that Pre-reperfusion IA-CSI was associated with decreased expression of inflammatory markers such as TNF-alpha, ICAM-1, and IL-1beta. This was further supported by a study by Kurisu et al. in 2016 which found that Pre-reperfusion IA-CSI was associated with decreased activation of the inflammatory cascade and improved cerebral microcirculation (32). Kurisu et al. also found that IA-CSI has a protective effect on the blood brain barrier through inhibition of Post-reperfusion aquaporin 4 surge. Improved blood brain barrier preservation was also demonstrated in a 2004 finding by Ding et al. which found that selective TH improves blood brain barrier preservation through decreased matrix metalloproteinase overexpression and marked reductions in cerebral edema (32, 33). Additionally, studies have shown that Pre-reperfusion IA-CSI treatment for AIS widens the therapeutic window for reperfusion to 2–2.5 h, which has the potential to significantly improve morbidity and mortality for AIS patients (28).

Several studies have also evaluated the effectiveness of selective TH through simultaneous IA-CSI and reperfusion therapy (25, 29, 30). These studies used slower, prolonged 20°C saline infusion rates at 0.6 ml/min for 10 min (25, 29, 30). Two of these studies achieved target temperatures of 33–34°C within 5 min, which was sustained up to an hour Post-reperfusion without vital sign abnormalities (25, 29). These studies reinforce the effectiveness of selective TH as adjuvant treatment with reperfusion therapy, also demonstrating significant reductions in infarction volume and improved neurologic preservation compared to systemic TH treatment or saline infusion at body temperature (37°C) (29, 30). One of these studies also found that improved neurologic preservation was maintained over 28 days postoperatively.

Post-reperfusion selective TH has also shown promise as a therapeutic intervention for AIS. In 2012, Ji et al. conducted a study introducing a novel, interrupted method for IA-CSI compared to traditional, continuous IA-CSI administration in order to counteract the effects of hemodilution (31). The interrupted IA-CSI technique consisted of tri-sectional infusions with 20 min no-infusion intervals between infusion sessions. Both methods of intervention demonstrated markedly reduced infarct volumes and cerebral edema, however, interrupted IA-CSI was found to drastically prolong hypothermia without evidence of hemodilution (31). In another study, Ji and colleagues assessed Post-reperfusion IA-CSI by comparing continuous IA-CSI with intra-carotid body temperature saline infusion at various latency periods (34). Their results found that in shorter periods, both methods significantly reduced infarct volume, but only IA-CSI was associated with decreased cerebral water content, reduced cell death mediator expression, and improved neurologic preservation (34). Further, Ji et al. found that during extended latency periods, only IA-CSI showed beneficial outcomes. This landmark study built upon the hypothesis that IA-CSI treatment may widen the therapeutic window for reperfusion in AIS patients, which has potential for tremendous reductions in AIS morbidity and mortality and set the precedent for future primate and human trials (34).

As an alternative to saline infusion alone, IA-CSI with alternative neuroprotective agents have also been studied. A study by Chen et al. evaluated the effects of local, cold low-dose albumin infusions as an alternative to saline infusion in AIS (35). Regional, low-dose 0°C human albumin was infused at a rate of 0.5 g/kg into rats with induced tMCAO was found to outperform local cold saline, local and systemic normothermic albumin; and local and systemic normothermic albumin in infarct volume reduction and neurologic function recovery (35). IA-CSI with magnesium sulfate has also been found to reduce infarct volume and enhance neuroprotection in AIS rats, further reflecting the synergistic effects of IA-SCI with other neuroprotective agents (35, 36).

The majority of studies on the utility of selective TH in AIS have used rodent models. This could partly be due to the ability of rodents to better tolerate deeper hypothermia compared to primates and ease of handling, therefore providing more convenient study conditions. However, the use of rodent AIS treatment models accompanies many limitations that limit its direct translation to the clinical setting, such as the differences in brain size (smaller brain size inherently results in faster cooling rates) and post-AIS molecular inflammatory cascades in rodents compared to humans (37). This, along with recommendations from the Stroke Therapy Academic Roundtable to study novel AIS treatment in large animals prior to human trials, has led to increased use of primate models in studying AIS therapeutic intervention in more recent years (37–39) (Table 2).

**Table 2:** Studies on large animal and non-human primate.

**Author**	**Species**	**Infusate/Volume**	**Infusion rate**	**Infusion duration**	**Time to TT**	**Brain temp**	**Core body temp**
Furuse et al. (40)	Canine	Ringer's solution 6.5°C (>1,000 ml)	38.9–43.4 ml/min	30 min	30 min	33.6°C	34.1°C
Caroff et al. (41)	Canine	Saline 4.5°C (515 ml)	20–40 ml/min	14.4 min	<5 min	23.8°C	37.2°C
		Saline 4.5°C (550 ml)	22 ml/min	25 min	<5 min	31–32°C	37.2°C
Wang et al. (42)	Rhesus monkey	Ringer's solution 0–4°C (100 ml)	5 ml/min	20 min	10 min	Cortex 34°C Striatum 33.9°C	37.1°C
Wu et al. (43)	Rhesus monkey	Ringer's solution 0–4°C (100 ml)+Alteplase (1.1 mg/kg)	5 ml/min	20 min	10 min	—	—
Cattaneo et al. (44)	Ovine	0.9% Nacl(−6°C)	—	180 min	180 min	33°C	−3°C
Mattingly et al. (45)	Swine	Extracorporeal criculation	—	36–150 min	<30 min	26°C	34°C
Fazel et al. (46)	Swine	cold air (−3 ± 2°C)/ —	40–50 L/min	50–60 min	1 h	33.7°C	37.3°C

*TT, Target Temperature*.

### Large Animal Models

The first study of selective TH for AIS in large animals was conducted in 2007 by Furuse and colleagues using endovascular intra-arterial infusion into the right common carotid artery of canines with tMCAO (40). Ringer's lactate solution at 6.5°C was infused at a rate of 3 ml/kg/min for a duration of 30 min using an angiographic catheter through the right femoral artery (40). The cooling rate of the right cerebral hemisphere was 4°C/30 min, with no significant increase in cerebral extracellular lactate concentration, although hemoglobin and hematocrit decreased significantly during perfusion, they trended toward recovery in the Post-perfusion period (40). On the contrary, cardiac output significantly increased during perfusion and insignificantly decreased during the Post-perfusion period (40). All canines were successfully cooled rapidly and survived without neurological deficits (40). A more recent tMCAO canine study conducted in 2018 by King and colleagues reinforced the promise of selective TH through endovascular cooling using a novel insulated catheter. King et al. showed that selective TH can be achieved quickly and safely and result in significantly smaller infarct volumes than without selective TH (47). Selective TH through endovascular cooling was further validated in AIS canines in 2019 by Caroff et al. through infusion of cold saline at 4.5°C at a rate of 22 ml/min for 25 min using a novel insulated catheter to minimize heat transfer (41). The ipsilateral hemisphere was effectively cooled while minimizing infusion volume (41). This technique resulted in significantly smaller infarct volumes compared to the control group, making strides toward clinical translation by demonstrating these positive effects in large animals using realistic infusion rates and volumes (41).

Selective TH has also been studied using swine and ovine models. Cattaneo and colleagues assessed IA-CSI with a novel balloon cooling catheter system through the common carotid artery in tMCAO sheep. This study demonstrated that this novel method could rapidly induce hypothermia in the ipsilateral cerebral hemisphere and may benefit AIS patients in combination with mechanical thrombectomy (44). Using a swine model, Mattingly et al. studied selective TH in swine with tMCAO using a dual lumen balloon occlusion catheter simultaneously with reperfusion. Mattingly and colleagues induced tMCAO for 3 h using an aneurysm clip, and the outflow catheter was placed in the thoracic aorta to allow blood to exit, become chilled, and then reperfused through an inflow catheter in the common carotid artery (45). Target temperatures of <30°C were reached on average within 15 min and resulted in markedly reduced infarct volumes (45). In addition, intranasal cooling systems have also been widely used in models of hypothermia in swine and ovine stroke models. The nasal anatomical location contributes to heat exchange with the cervical blood and cerebrospinal cord fluid flowing into the brain. The nasal cooling was induced by either a cold water circulating device or coolant mist diffusion. For example, Abou-Chebl et al. assessed the utility of intranasal cooling using a coolant device that sprays perfluorocarbon-oxygen mist into the nasal cavity, where the coolant evaporates and absorbs heat from the tissue through direct conductive and indirect hematogenous mechanisms (48). Intranasal cooling has also been tested by Bakhsheshi et al., who used mechanical tubes to blow cooled air into the nasal cavities, which they theorized cooled the brain selectively due to the cavernous sinus's proximity to blood in the internal carotid artery and cerebrospinal fluid in the basal cistern (46). Both of these intranasal methods are advantageous because they achieve local brain cooling while being minimally invasive, portable, and user friendly without additional extensive training (46, 48). In addition, a new study using a closed-loop system has shown the effectiveness and safety in porcine models, which also prevented local and systemic adverse events (46, 48–51). Since the catheter is introduced only in the left nostril, the preferential decrease in brain temperature radiates on the left side. This effect may help determine the priority of cooling on one side in local neurological disorders such as stroke. The major limitation of swine and ovine experiments is the capability to perfectly mimic the physiology and anatomy of humans due to the presence of a larger nose-to-brain volume. Therefore, Non-human primate and human testing is crucial to ultimately prove cooling efficacy (Table 2).

### Non-human Primate Models

Selective TH has also been studied in rhesus monkeys with induced tMCAO (42, 43, 52). In a 2016 study by Wang and colleagues, 0°C Ringer lactate solution was used to induce selective TH in rhesus monkeys with tMCAO (42). The volume of infusion was calculated in proportion to the infusion volume and body weight of rats used in Ding's earlier studies, and the total infusion volume was 100 ml (42). When IA-CSI was infused at 5 ml/min, 0–4°C, Ringer lactate solution reached mild cerebral hypothermia (<35°C) within 10 min; much faster than whole-body infusions using the same protocols (42). Importantly, no significant fluctuations in rectal temperature, hematocrit, cerebral blood flow velocity, or cerebrovascular reactivity were observed during or after reperfusion. In addition, no cerebral edema, new infarction, hemorrhage, or vasospasm were observed, which further validated the safety, feasibility, and efficacy of IA-CSI (42). A 2020 study by Wu et al. combined alteplase with the above-mentioned IA-CSI model for IA thrombolysis, found that those with full or partial reperfusion significantly reduced the volume of infarction, alleviated neurological dysfunction, and improved upper extremity motor dysfunction in both acute and chronic stages (43). However, no further neuroprotective was observed in monkeys without reperfusion (43).

### Clinical Evidence for Selective Hypothermia and Potential Applications in Stroke

The progression to larger animals from rodent models and subsequent successful outcomes are encouraging evidence supporting clinical translation of selective TH in the treatment of AIS. However, several quandaries regarding physiologic differences between animals and humans remain that cannot be addressed using animal models (38). Given the promise IA-CSI has shown in the treatment for AIS in both rodent and large animal studies, clinical trials of selective TH have finally begun (Table 3).

**Table 3:** Clinical trials.

**Author**	**Infusate/Volume**	**Infusion rate**	**Infusion duration**	**Time to TT**	**Brain temp**	**Core body temp**	**Infarct volume**
Choi et al. (24)	Saline 4–17°C(330 ml)	33 ml/min	10 min	<10 min	−0.84°C(JVBT)	−0.15°C	—
Chen et al. (53)	Saline 4°C(350 ml)	Before reperfusion 10 ml/min	Before reperfusion 5 min	—	−2°C	−0.1°C	—
		After infusion 30 ml/min	After infusion 10 min				
Wu et al. (54)	Saline 4°C(350 ml)	Before reperfusion 10 ml/min	Before reperfusion 5 min	—	—	36.5°C	Reduce
		After infusion 30 ml/min	After infusion 10 min				
Poli et al. (55)	Coolant gas/—	60 L/min	1 h	1 h	—	—	—
Abou-Chebl et al. (48)	Coolant gas/—	80 L/min	1 h	1 h	−1.4°C	−1.1°C	—
Ferreira et al. (56)	Circulating cold water (0–2°C)/ —	1.51 ± 0.36 L/min,	24 h	9.5 h	−2.5°C	36.0°C	—

*TT, Target Temperature; JVBT, Jugular Venous Bulb Temperature*.

Choi and colleagues (24) were the first to evaluate the safety and feasibility of selective TH in a study of 18 patients in 2010. Patients were equally divided into two groups. The first group was used to establish the safety of infusion: 12–17°C cold saline was injected into one side of the internal carotid artery at a rate of 33 ml/min for a duration of 10 min. The second group, using the same protocol, received infusions of lower temperature saline at 4–7°C, and although the bulb temperature decreased by an average of 0.84°C, the core temperature only dropped by an average of 0.15°C. The biggest drawback of this experiment is that it could not measure the temperature of the brain parenchyma directly as was done in animal studies. Since the jugular venous bulb drains blood from the entire head, the decrease in target parenchymal tissue was likely much greater than the measured 0.84°C (57, 58). However, in a subsequent 2013 study, the team used mathematical models to predict changes in brain parenchymal temperature more accurately. They utilized a previously established biophysical mathematical model, which estimated that a 10 min infusion time would decrease the temperature of ipsilateral anterior circulation territory by about 2°C. It takes less time to reach a temperature comparable to the systemic hypothermia temperature (59).

A study by Chen et al. in 2016 was the first pilot study of selective TH in AIS patients. Twenty six patients with large vessel occlusion eligible for mechanical thrombectomy were enrolled within 8 h of AIS onset (53). Each patient received IA-CSI with 4°C cold isotonic saline in conjunction with reperfusion treatment into the ischemic region. Although the study did not measure venous outflow temperatures, the combination of Pre-reperfusion and Post-reperfusion IA-CSI was estimated to decrease the temperature of ischemic cerebral tissue by at least 2°C during infusion of the 4°C isotonic saline and systemic temperatures were mildly reduced (53). Importantly, no obvious complications related to IA-CSI occurred (53). This landmark study demonstrated that IA-SCI treatment with cold saline is a safe and practical method to employ in patients with AIS (53).

Following the success of the 2016 pilot study, a larger prospective cohort study was launched in 2018 by Wu et al. to evaluate the safety and efficacy of IA-CSI in patients undergoing mechanical thrombectomy compared to mechanical thrombectomy without IA-CSI (54). Using the same pre- and Post-reperfusion infusion protocol, the Wu et al. study found that IA-CSI reduced infarct volumes by an average of 19.1 ml, based on Non-contrast CT 3–7 days Post-intervention. Further, this study demonstrated that IA-CSI promoted functional independence measured at 90 days Post-procedure compared to patients who received only thrombectomy therapy, although this difference was not statistically significant (54). However, patients who received both IA-CSI and reperfusion therapy had lower Alberta Stroke Program Early Computed Tomography Scores and poor collateral circulation at baseline, thus it is likely that the neurological function retention provided by IA-CSI may be stronger than shown in this study (54). Nonetheless, these results still provide valuable data for future follow-up clinical trials. Currently, two clinical trials are underway, being conducted by Tokairin et al. and Wu et al. to further evaluate the utility of IA-SCI combined with mechanical thrombectomy in AIS patients (60, 61).

Due to the Non-invasive and convenient nature of the nasal cooling technique for TH, this method may be a favorable possibility for clinical translation. Twenty stroke patients who received neurological monitoring and treatment in a neurocare unit underwent intravenous cold systemic cooling or intranasal cooling at a rate of 60 L/minute in a 1:1 random manner (55). While systemic cold infusions achieve brain cooling faster than intranasal infusions, their effects on systolic arterial pressure, mean arterial pressure, intracranial pressure, and cerebral perfusion pressure raise concerns about the safety of both cooling methods. A similar study in stroke patients in intensive care investigated the feasibility of cooling the brain within 1 h of nasal cooling with a coolant flow rate of 80 L/minute (48). However, the core temperature dropped by 0.6°C. Transient hypertension was the only adverse event in 1 patient, for whom intranasal cooling was stopped.

The administration of fluorocarbon-rich gasses (48, 55, 62) and oxygen (46, 49–51) do not allow precise temperature control, and safety concerns remain (63). So, the closed-loop system for circulating cold water was explored. A prospective, Non-randomized, interventional clinical trial involving five patients with severe traumatic brain injury (56). Interventions included inducing and maintaining selective brain cooling for 24 h by positioning the catheter in the nasopharynx and circulating cold in a closed loop arrangement within the catheter. Using counter-warming to keep the core temperature at ≥35°C. Studies have demonstrated that this method is safe and effective in humans. However, due to low recruitment rates, investigators chose to terminate the study early. Although the sample size is small, five patients are sufficient to determine the outcome of the Pre-specified outcome and have the potential to demonstrate the clinical and physiological benefits of the technique.

## Other Novel Selective Hypothermia

More recently, a novel technique of brain cooling via the internal jugular vein (IJV) has been explored as a more convenient, faster, and economically savvy method of inducing selective TH. Duan et al. demonstrated the effectiveness of IJV administration in rats with tMCAO, by comparing IJV cooling with internal carotid artery cooling, both of which were infused with 6 ml of 0°C isotonic saline for 30 min. Findings demonstrated similar efficacy and neuroprotective benefits to those provided by intracarotid hypothermia (60).

The main advantage of using the IJV as a cold infusion channel is the ease of entry. The IJV is the most common site for central tube placement, a common bedside procedure. Thus when an acute stroke occurs, a tube for cold infusion could theoretically be placed with ease in the emergency room within minutes. In contrast, the carotid artery or direct access to the infarction site can only be accessed under surgical conditions. Additionally, theoretical models have shown that when the two carotid arteries are at sufficiently different temperatures, they tend to lose heat through IJV reverse cooling (64). Therefore, selective TH through IJV cold infusion may be cooled by the countercurrent of the carotid blood. Although research must be done to demonstrate the efficacy and safety of selective TH through IJV cold infusion, the use of the IJV for infusion could be instrumental in providing early AIS treatment to minimize brain damage and maximize functional preservation.

Hypothermia has also been found to enhance the efficacy of other neuroprotective agents (65). Combination therapies involving hypothermia and adjunctive drugs, including phenobarbital, topiramate, erythropoietin, xenon, magnesium sulfate solution, have been reported to be more effective than TH alone (36, 66–69). In a 2017 preclinical study, Wu and colleagues found that while IA-CSI or dihydrocapsaicin alone achieved acceptable cooling and neuroprotection, their combined administration further reduced brain temperature and infarct volume and improved ischemic neurological deficit. The combination of physical and pharmacological hypothermia positively affects energy metabolism, oxidative stress, apoptosis, blood-brain barrier integrity, and inflammatory responses (70, 71). In 2021, Byun et al. demonstrated that the combination of carnosine and hypothermia was neuroprotective in a neonatal rat model of hypoxic-ischemic brain injury (72). In the same year, a study conducted using neonatal rats with hypoxic brain injury used combined treatment with carnosine and hypothermia, which resulted in reduced brain damage (73). These studies provide the possibility to explore more diverse treatments for neurological diseases.

## Perspective and Prospective

While research on selective TH has made major strides in the last two decades, there is still an urgent need for progress in clinical translation prior to clinical implementation as an adjuvant treatment for AIS. Studies completed to date have utilized a variety of target temperatures, infusion durations, induction times, and methods, and thus a standardized approach for selective TH has not yet been established. Further, while studies have demonstrated safety and efficacy of this treatment, this procedure has the potential for serious adverse effects resulting from deep hypothermia or excessive infusion. In order for selective TH to gain clinical approval, depth, duration, and treatment window, as well as type of infusion with or without other medicines must all be determined and optimized. Future studies should focus on optimizing these variables in clinical trials so that a standard operating procedure may be established.

The neuroprotective benefits of selective TH have been well demonstrated in preclinical models and its safety and feasibility have been well established in clinical models of AIS. It is worth mentioning that the technical skills and equipment required for selective TH treatment are no different than those to perform a thrombectomy, and there are no obvious financial barriers to employing selective TH in conjunction with reperfusion treatment. Therefore, selective TH shows immense promise as an adjuvant technique to improve acute and long-term outcomes in patients who have suffered AIS.

## Author Contributions

XW, AW, SK, and NC wrote and edited the manuscript. XG and YD were also involved in drafting the manuscript and revising it critically for important intellectual content. All authors contributed to the article and approved the submitted version.

## Funding

This study was supported partially by the National Natural Science Foundation of China (82072549, 81871838, 82001277, and 82101436), the Youth Scientific Research Incubation Program of Beijing Luhe Hospital, Capital Medical University (LHYY2021-JC04), the Beijing Tongzhou District Financial Fund, and the Science and Technology Plan of Beijing Tongzhou District (KJ2022CX033).

## Conflict of Interest

The authors declare that the research was conducted in the absence of any commercial or financial relationships that could be construed as a potential conflict of interest.

## Publisher's Note

All claims expressed in this article are solely those of the authors and do not necessarily represent those of their affiliated organizations, or those of the publisher, the editors and the reviewers. Any product that may be evaluated in this article, or claim that may be made by its manufacturer, is not guaranteed or endorsed by the publisher.
